# Lifesaving skills training in schools – A qualitative study to explore students, teachers, and parent’s perceived opportunities and challenges

**DOI:** 10.1186/s12889-023-15284-9

**Published:** 2023-02-27

**Authors:** Natasha Shaukat, Daniyal Mansoor Ali, Mehtab Jaffer, Zeerak Jarrar, Naela Ashraf, Sheza Hassan, Ali Azim Daudpota, Muskaan Abdul Qadir, Aly Hamza Khowaja, Junaid Razzak

**Affiliations:** 1 Centre of Excellence for Trauma and Emergencies, Aga Khan University, Stadium Road, P.O. Box-3500, Karachi, 74800 Pakistan; 2 School of Nursing & Midwifery, Aga Khan University, Karachi, Pakistan; 3 Aga Khan University Medical College, Karachi, Pakistan; 4 Department of Emergency Medicine, Weill Cornell Medicine, New York, USA

**Keywords:** Bystander intervention, Lifesaving skills, School Children, Education, Qualitative research

## Abstract

**Objective:**

The objective of this study is to explore the perception of teachers, parents and students’ regarding implementation of a school-based lifesaving skills program and help predict potential barriers and solutions.

**Methods:**

This qualitative exploratory study was conducted in Karachi, Pakistan, from December 2020- to October 2021. We included students, teachers, and parents of secondary (grades VIII, IX, and X) and higher secondary level students (grades XI and XII) in Karachi, Pakistan's public and private schools and colleges. We selected one public, two semi-private, and two private schools. We recruited students, teachers, and parents through convenience sampling. We conducted fifteen focus group discussions (FGDs) with the students, six FGDs with the teachers, and eighteen in-depth interviews (IDIs) with parents. We transcribed the data from audio recordings and translated it into the English language. Finally, we manually analyzed the data using thematic analyses.

**Results:**

This study found that bystanders' main barriers to performing lifesaving skills are lack of knowledge, fear of legal involvement, fear of hurting the patient by incorrect technique, lack of empathy among community stakeholders, and gender bias. However, the participants had a positive and supportive attitude toward implementing lifesaving skills training in schools. They suggested starting student training in the early teenage years, preferred medical staff as trainers, and suggested frequent small sessions in English/Urdu both or Urdu language and training via theory and practical hands-on drills. Furthermore, the training was proposed to be integrated into the school curriculum to make it sustainable. Finally, the government needs to support the program and make the legal environment more conducive for bystanders.

**Conclusion:**

This study identified the significant barriers to performing lifesaving skills in an emergency in a low- and middle-income country (LMIC). The participants supported implementing a national lifesaving skills program in schools and colleges. However, the participants expressed that support is needed by the government for sustainability, integrating lifesaving skills into the school curriculum, providing legal support to the bystanders, and creating awareness among the general public.

## Introduction

Out-of-hospital cardiac arrest (OHCA) and injuries are the leading causes of death worldwide [1]. Both disproportionately affect low- and middle-income countries and outcomes of both can be improved significantly through early bystander intervention [2–4].

Evidence from high income countries shows a greater likelihood of intervention by those who are trained in lifesaving skills [5, 6]. Additionally, the ideal population for bystander CPR training is primary and secondary school children. World Health Organization (WHO) endorsed the bystander CPR training curriculum for students [7–9] with increased frequency of school-based programs in high- and middle-income countries [10–14].

Research to assess students, teachers, and parents knowledge, attitude, and perception regarding bystander CPR and stopping the bleed training have been conducted primarily in high income countries. A study of high school graduates in Australia showed high support for mandatory CPR training in schools [15]. Another study explored barriers to implementing CPR training in Danish secondary schools. The teachers had insecurity about their skills, and believed external instructors were needed for training students, as CPR requires extra-ordinary skills [16].

Few studies were conducted in low-and middle-income countries. A study among Chinese university students identified cultural barriers and feeling embarrassed in providing CPR [17]. Studies from Ghana, and Lebanon identified lack of skills and fear of hurting victim as major barriers to provide CPR. The study suggested hands on trainings to build bystanders confidence and willingness [18, 19].

The objective of this study is to explore the perception of teachers, parents and students’ regarding implementation of a school-based lifesaving skills program and help predict potential barriers and solutions.

## Methods

### Study setting

Pakistan is the world's fifth-most populous country, with four provinces: Punjab, Khyber Pakhtunkhwa, Sindh, and Balochistan. Karachi is a metropolitan city located in Southern Sindh, with an estimated 22 million population. Education in Pakistan is decentralized, with powers delegated to the provinces. Education is free and compulsory for all children between the ages of five to sixteen. The education system in Pakistan is divided into six levels: preschool (ages three to five years); primary (grades one through five); middle (grades six through eight); secondary (grades nine and ten); higher secondary (grades eleven and twelve); and university programs leading to graduate and advanced degrees. The education system of Pakistan is comprised of 260,903 institutions and is facilitating 41,018,384 students with the help of 1,535,461 teachers. The system includes 180,846 public institutions and 80,057 private institutions. Hence 31% of educational institutes are run by the private sector, while 69% are public institutes [20]. Karachi has both public and private educational institutes from primary to university levels. This study was conducted in Karachi in six (public, private, and semi-private) schools/colleges.

### Study design

A qualitative exploratory design was used for this study, which provides an opportunity to explore and understand in-depth about a single concept and assists in describing human experiences.

### Study population

The study population comprised students, teachers, and parents of secondary (grades VIII, IX, and X) and higher secondary level students (grades XI and XII) in public and private schools and colleges of Karachi, Pakistan.

### Eligibility criteria

Students, teachers, and parents of secondary (grades VIII, IX, and X and aged 13–16 years) and higher secondary students (grades XI and XII and aged 17–20 years) in public and private schools and colleges of Karachi who were willing to consent were included in the study.

### Sampling technique

We used Convenience sampling to select the study participants. We contacted and emailed various schools to invite them to participate in the study. We included those schools that gave us a positive reply. We discussed the study's objectives in a meeting with the school administration. The school management presented the study to the teachers and students. The school administration sent consent forms home with students to inform parents and get their permission to participate in the study. The schools asked teachers to self-nominate for the study. The schools chose groups of students and teachers for the FDGs and provided us with the participant lists. The schools arranged dates, times, and venues for the interviews. For the parent interviews, we called the parents from the contact lists provided by schools and scheduled them at days and times of their convenience.

### Sample size

We selected one public, two semi-private, and two private schools. We conducted fifteen FGDs with the students and six FGDs with the teachers. There were eight to twelve participants in each student FGDs and five to seven participants in the teacher's FGDs. In addition, we conducted eighteen semi-structured interviews with the parents of grades (IX, X, XI, and XII).

### Data collection methods

We collected data during the COVID-19 pandemic (December 2020–October 2021), when most schools and colleges offered online or hybrid classes. Similarly, we used a hybrid approach (online and in-person focus groups) to collect our data. The school administration scheduled FGDs during free periods. Furthermore, except from the research team and study participants, no other people were permitted in the room during the FGD. During the COVID-19 pandemic, we conducted interviews on Zoom when the schools and institutions were closed. The school administration formed the groups. They gave us the email addresses of all participants and scheduled the interviews' times and dates. For the parent interviews, those who agreed to participate were interviewed telephonically at days and times of their convenience. We conducted interviews in both English and Urdu language. Each interview lasted between 45 and 60 min. We recorded the discussions digitally. Additionally, we kept field notes and reflective logs.

### Data collection tools

The interview guide consists of initial demographic information section (age, gender, grade, qualification), and the knowledge and perceptions of bystander training. The interview guides were developed in English and Urdu languages.

#### For students

The research team developed semi-structured interview guides for focus group discussions with students. This guide explored students’ knowledge, attitude, and perceptions (KAP) towards CPR and bleeding control training. Further, we inquired about their understanding of CPR and hemorrhage control, willingness to help in an emergency, ability and perceived barriers to performing CPR and hemorrhage control, and barriers and facilitators in training school children as bystanders.

#### For teachers

Semi-structured interview guides for teachers focused on their knowledge, attitude, and perceptions towards CPR and bleeding control training. In addition, we specifically explored the effective ways of delivering CPR and bleeding control training in schools, duration of courses, language of the courses, mode of delivery, sharing of information, barriers, and facilitators in training school children as bystanders.

#### For parents

Semi-structured interview guides for parents explored their knowledge, attitude, and perceptions towards CPR and bleeding control training. We inquired about their current understanding of CPR and hemorrhage control, willingness to help in an emergency, and perceived barriers to perform CPR and hemorrhage control, effective ways of learning CPR and hemorrhage control training, their beliefs about children's ability to learn and perform CPR and hemorrhage control, their concerns in children receiving CPR and haemorrhage control training in schools.

### Data management and analysis

The data from audio recordings were transcribed and translated into English by research assistants and medical doctors with experience in translation who were well-versed in both Urdu and English. We manually analysed the data using thematic analyses as outlined by Braun and Clarke [21]. We read and re-read the transcripts to get familiar with the data and the patterns in the data. After generating initial codes, we combined these codes to generate initial overarching themes. The themes were then reviewed against the codes and the entire data set.

Further, we defined and named the themes to produce the report. An experienced qualitative researcher (MJ) acted as a coresearcher at all stages of the analytic process to ensure credibility. NS and MJ analyzed the data independently. In addition, MJ reviewed all the coding and themes, thus supporting a reflexive process.

## Results

One hundred seventy students participated from private and public schools and colleges. Of these, 148 were from private and 24 from public schools. Fifty teachers participated, of which 42 were from the private sector and eight from the public sector. Eighteen parents were interviewed Table 1.

**Table 1:** Characteristics of study participants

Characteristics	Total Number (N)
**Students**	172
Sector	
*Private*	148
*Public*	24
Gender	
*Male*	55
*Female*	117
Grades	
*Grade VIII*	10
*Grade IX*	34
*Grade X*	34
*Grade XI*	38
*Grade XII*	56
**Teachers**	50
Sector	
*Private*	42
*Public*	8
Gender	
*Male*	15
*Female*	35
Grades	
*Secondary*	29
*Higher Secondary*	21
**Parents**	18
Sector	
*Private*	18
*Public*	-

Overall, four main sub-themes emerged have emerged, as shown in (Table 2).

**Table 2:** Emergent themes and sub-themes

Themes	Sub-Themes
Community Barriers in helping the injured victims	➢ Lack of knowledge and fears/anxieties ➢ Fear of Legal involvement ➢ Lack of empathy among community stakeholders ➢ Gender Bias ➢ Fear of hurting the patient by incorrect technique
Students as life savers	➢ Physical strength ➢ Confidence and competence ➢ Parental and family Permission/agreement ➢ Effect on student’s studies
Elements of Training program for the students	➢ Willingness for training ➢ Student’s age-group for training ➢ Type of trainers (Doctors or Teachers) ➢ Mode and Strategies for implementing training and opportunities ➢ Duration, ratio and language of trainer
Implementation of national-level lifesaving skills program	➢ Support from Government and Senior Leadership ➢ Integration into the curriculum ➢ Legal support and protection ➢ General public awareness and marketing

### Theme 1: Community Barriers in helping the injured victims

Most participants shared the barriers, fears, and apprehensions due to which people do not come forward and help in emergencies. Five-main sub-themes emerged are: Lack of knowledge and fears/anxieties, fear of legal involvement, lack of empathy among community stakeholders, and gender Bias

Fear of hurting the patient by incorrect technique

### Sub-Theme 1.1: Lack of knowledge and fears/anxieties

Lack of knowledge and awareness about what to do and how to provide help to the patient in case of an emergency was the most commonly cited barrier among participants. Participants said that they do not provide help in an emergency since they are unaware of any lifesaving techniques or strategies.

One of the students said.

“CPR is done to help the patients. Like when someone has an abnormal heart beat so we punch here to make the heartbeat normal” (Participant 02, student FGD 06)

One of the parents said

“See I am a housewife. I don’t know how to do it. There is awareness issue. You will only apply if you have the knowledge”- (Parent IDI 06).

Some of them heard about CPR in the media or Facebook (FB) but no such experience in their personal life. They were unsure if the CPR technique would work.

“I have heard people blowing out in patients mouth to revive their lungs. But i don't know if it works”. (Parent IDI 05)

A teacher said

“We have heard this term but we do not have much knowledge about it” (Participant 01, Teachers FGD 03)

### Sub-Theme 1.2: Fear of Legal involvement

Fear of involvement in a police case was the most commonly reported fear by the majority of the participants. People were afraid to get their names involved in legal matters. Most participants said they did not want to get involved in legal issues when their intention was just to help the victim. A student said.

“If the police is going to be involved, they will include us in the investigation.”( Participant 03,Students FGD 07)

Fear of legal involvement was so overwhelming that even if the people wanted to help, they would not come forward, as they wanted to avoid getting involved in any of the legal issues.

“Actually, people here are afraid from law and enforcement agency. What if they put your name in first incident report (FIR)? These things in society fear people to help assist” – (Participant 05, Teacher FGD 01)

A parent said

“If god- forbid there is a major situation occur then people get scared of it that if anything goes wrong our law enforcement agencies will not support us.” – (Parent IDI 17)

### Sub-Theme 1.3: Lack of empathy among community stakeholders

Participants said that now people have become self-centered if they witness an accident. So, if a situation arises, people do not consider the victim as one of their own. The students added that in such a situation where people witness an accident, they would instead take pictures selfies, make videos, and post them on social media rather than come forward and help the victims.

One of the students said

“People nowadays are more interested in making videos and then uploading it on social media to get famous” -( Participant 05, student FGD 08)

Participants also talked about fast passed life and time factor. They felt that the dilemma is that people are very busy in their lives and do not have time to help others. Therefore, they will not stop to check on the patient and continue their journey un-bothered since they are not related to the victim.

One of the teachers said

“People prioritise their time so that they think they will waste time getting caught up in the situation. Also, they at times cannot feel for that person (victim) suffering” -( Participant 03, Teachers FGD 06)

One of the parents said

“People are very selfish now a days. This is what I have noticed. They save themselves that why should we get involve with others. Selfishness” (Parent IDI 17)

### Sub-Theme 1.4: Gender Bias

Some participants mentioned they would hesitate to help due to gender-based, cultural barriers. Women feel embarrassed to come forward and help if the victim is a male.

A student said

“If there is a girl and she knows what to do still she will not come forward because there are men in crowd”- (Student 02, FGD 07)

*Teachers shared*

“Gender difference could be a barrier in performing CPR. Being of the opposite gender as that of the victim’s would be the barrier in this case” (Participant 02, Teacher FGD 01)

Another teacher added females will not get permission from their families to come forward in such a situation.

“Would they be comfortable with men performing CPR on women and vice-versa? Would they even allow females there to learn CPR? So there will be many challenges. “ (Participant 05, Teacher FGD 03)

A parent said, that there would be hesitation in helping the opposite gender. However, he did acknowledge that this is not an issue in the western world.

“See gender bias is the first thing. If a man is dying, a woman cannot help him and when a woman is dying man is scared to approach. Abroad I have seen people helping each other.”- (Parent IDI 11)

“Being a woman, yes, I would be careful moving through a crowd”- (Parent IDI 09)

### Sub-Theme 1.5: Fear of hurting the patient by incorrect technique

Many participants expressed fear that they will hurt the patient if they perform CPR in real life. In addition, people expressed their reluctance to cause damage to the patient by either pushing too hard or performing it in the wrong way.

A student believed that

“For CPR I have heard you need to press on the chest and that could make some problem in the person’s cardiac issues and you could also hurt them”- (Participant 07, Student FGD 08)

One of the teachers shared her concern that she is really unsure about how to perform the procedure correctly. She felt that this might hurt the victim as she didn’t know how to correctly attempt the CPR.

“*We don’t know the right time and place. Maybe we do wrong instead of doing it correctly. What if I pressed more hardly?” (Participant 02, Teachers FGD 04).* The participant looked concerned while expressing her uncertainty.

One of the parents said

“I think lot of people have, umm, fear of something going wrong. I know, like I have fear of pushing too hard and breaking the ribs” -(Parent IDI 06)

### Theme 2: Students as lifesavers

The participants shared their views, opinions, and concerns regarding training students as lifesavers. The four main sub-themes that emerged are: physical strength, confidence and competence, parental and family permission/agreement, and effect on student’s studies.

### Sub-Theme 2.1: physical strength

Most participants believed gender and physical strength were not obstacles to learning and performing CPR. Most participants said the students especially female students had good physical strength to perform such skills. A student explained the physical strength of women by comparing it with childbirth.

“If a woman can tolerate the pain of breakage of 21 bones when giving birth, her physical strength is more than the others. If she can bear this, she can do anything”- (Participant 05, Student FGD 05)

A teacher said

“I think children in middle school and above are physically fit enough to do this” (Participant 3, Teacher FGD 4)

A parent had the same confidence in his daughter's skills as his son. He said, “*I don’t think my daughter lacks physical strength*” (*Participant 01, Student FGD 10).*

### Sub-Theme 2.2: confidence and competence

Parents and students felt a lack of confidence could hold back the children. The children might lack confidence initially, but with time, training and experience they will gain the confidence to perform these skills. Some students also felt less confident. They attributed it to; lack of adequate knowledge, not performing correctly and making mistakes, and fear of hurting people. Few students’ perspectives were.

“I can make mistake” -( Participant 02, Student FGD 03)

“I don’t want to take risk”- (Participant 05, Student FGD01)

“I am not sure about performing it on someone else”-(Participant 04, Student FGD 05)

A Parent said

“Probably not the first time. But after that she will be confident and knowledgeable about the actual process” -(Parent IDI 5)

### Sub-Theme 2.3: parental and family permission/agreement

Few teachers shared their concerns that the children will not be given the responsibility in the presence of older family members. A teacher said.

“My concern is that it is impractical for the children because even something happens at home, No one at home will allow children to do.” (Participant 05, Teachers FGD 07)

Most parents were encouraging and said they would allow their children to save someone's life if they got the opportunity. They would feel happy and proud if their child can save someone's life.

“I think I would allow my child to do that, in fact I would appreciate it if my child could be of help to someone.” (Parent IDI 16)

“Life and death are in the hands of Allah but if he can help to save lives like doctors it is his responsibility to help people.” (Parent IDI 14)

However, few parents felt uncomfortable in permitting their children to take charge in such situations. A mother was worried about her child being only son and said her child would need permission from the father.

“This is something his father can tell because I only have one son” (Parent IDI 06)

“He cannot go far. If something happens in our neighbourhood, then he can help" (Parent IDI 15)

### Sub-Theme 2.4: effect on student’s studies

Some participants shared their concerns that the children's studies might get affected. They provided justifications that, the children were already overwhelmed with such busy timetables, upcoming exams, and introducing such training might hinder their academics. Some of the students felt uncomfortable due to the heavy academic load. They suggested conducting the training in free periods so that their studies are not compromised. A student said.

“Ma’am if you give us training in one period that is fine but if you want us to study more about it, then we have our own studies and they will get affected.” (Participant 06, Student FGD 09).

A parent said

“My son is in matric and the education load on them is way too much. They have their exams coming up it will be difficult for them to manage” (Parent IDI 05)

### Theme 3: elements of training program for the students

All participants supported training students with lifesaving skills in schools in Pakistan. They talked about the inter-related components needed for training students with lifesaving skills in schools**.** Six main sub-themes are: Willingness for training, students age group for training, type of trainers (Doctors or Teachers), mode and strategies for implementing training and opportunities, duration, ratio and language of trainer**.**

### Sub-Theme 3.1: willingness for training

Almost all the participants had a positive attitude and showed a willingness to train for lifesaving skills and felts everyone should know these skills. They supported imparting lifesaving skills to students in the schools. They suggested that it would be suitable to train those on the fore-front, i.e., students, teachers, administrative staff in schools, police officers, and working force. Almost all the parents felt that it was good to equip children with these skills since anyone can face an emergency.

A student said

“Everyone should know that in case of emergency when an ambulance is on the way, what they can do to help” (Participant 04, Students FGD 16)

A teacher shared her thoughts

“It will help them a lot that they will know what to do when there is an accident, what and how to do.” (Participant 02, Teachers FGD 06)

Few parents shared

“I think traffic police is very important on roads. Similarly, in institutes teachers and staff are main people, train them also” -(Parents IDI 10).

“It should be taught and children should know how to perform CPR. Children should be able to deal with these situations. Moreover, in situations like these, it is very difficult to find a doctor so children should be enabled to perform CPR”. (Parent IDI 05)

### Sub-Theme 3.2: student’s age-group for training

Most participants suggested that this training should begin early on in life. The parents suggested it was suitable, to begin with training students in grade 9^th^ and above in terms of age group.

**“**At least 13 years of age”- (Participant 5, Student FGD 09)

Teachers also suggested to train the secondary school's children.

“We should start from secondary classes, because this is when a child begins to understand complex concepts like these. What is taught early in life usually remains with the person forever.” (Teachers FGD 1)

Parents shared their thoughts

“Girls and boys of class 9^th^, matric and also inter should be taught” (Parent IDI 03)

“The kid has to be mature that I can’t really specify an age. Over 15 would be good, I think". (Parent IDI 11)

### Sub-Theme 3.3: Type of trainers (Doctors or Teachers)

Most of the participants trusted doctors or medical professionals as better trainers, since they were the subject experts. Students had a mix-response; some were comfortable learning from the doctors, while some supported their teachers in this regard.

“We are not comfortable with anyone other than doctors.” (Participant 2, Students FGD 4)

“This will be best. They (teachers) already teach us so we will be able to learn theory and practical combined in this way.” ( Participant 4, Students FGD 9)

Most teachers supported training teachers to learn lifesaving skills, but there was a strong opposition for curriculum teachers as trainers. They felt that if this training program was to be implemented, then only newly hired teachers, physical education teachers, science/biology teachers should conduct this training for the students in the schools. They suggested recruiting new teachers designated explicitly for these training, increasing the number of Physical Education (PE) teachers so that the curriculum teachers are not burdened with this additional work.

“Sports teachers should be trained; the number of sports teachers should be increased so that the knowledge can be delivered to students better. The curriculum teachers are already occupied with exam preparation, syllabus completion, and result making, amongst other things. I do not think burdening these teachers with this extra task would be a good decision”. (Participant 2, Teacher, FGD 1)

“We should have designated teachers for this course, as curriculum teachers already have plenty on their plates. While curriculum teachers should also be taught these skills, separate, qualified instructors should be hired for the job of educating the students.”- (Participant 3, Teachers FGD 1)

“The PE teachers are also separate from curriculum teachers. This responsibility should not fall on the shoulders of curriculum teachers”- (Participant 4, Teacher’s FGD 1)

Some teachers suggested that science teachers would be the appropriate choice amongst the curriculum teachers.

“Those who already have a science-based degree such as medicine, biochemistry, or biology, would be better suited to teach this content”- (Participant1, Teacher FGD 04)

“I think science or biology teachers can give proper awareness as they know better about human body as compared to me. I am an accounts teacher. They can better function and structure the training because they have more awareness of body parts as they teach about it.” (Participant 2, Teacher FGD 04)

The trust in teachers as trainers was low for most of the parents.

“Do you think that we have that kind of teachers available who are capable of teaching children CPR effectively. The doctors should make teams and then train the children”- (Parents IDI 15)

“Doctors should teach them. Teachers’ job is to teach. I think students will learn better from doctors. I think students will trust and learn more from the doctors” -(Parents IDI 8)

### Sub-Theme 3.4: mode and strategies for implementing training and opportunities

Almost all the participants suggested conducting one-on-one, face-to-face sessions for these training. They felt lectures were appropriate for teaching the theory, and practical/hands-on drills were suitable for the skills session.

Students shared their thoughts

“In-person would be the best way because online lectures are boring. We can show proper demonstrations in the in-person sessions as well.”- (Participant 3, Students FGD 9)

“Give everyone a chance to practice on mannequins”-(Participant 2, Students FGD 8)

Teachers said that not all students have access to the internet and smartphones. Teachers were worried that a large chunk of students, especially those in rural areas/low-socio-economic strata, would be left out in case of online teaching due to lack of resources (electricity, internet, smartphones).

“Online access appears to be the biggest problem, as in a class of more than 20, hardly 5 have access to the internet. Many of the students will miss out if these sessions are conducted online”- (Participant 5, Teacher FGD 03)-The teacher looked worried while he expressed his thoughts.

“We teach rural schools and our students do not have the resources. Only 2 or 3 students in a class have cell phones or other handheld devices. These are kids who at times cannot even afford school fees. Sir this will not work, it is not a valid option for our schools. Here in our areas, the only option seems to be that the teachers themselves conduct in-person sessions”. (Participant 2, Teachers FGD 03)

Parents also favoured face to face teaching

“Online is not good. Most of the children do not have WhatsApp. It is very difficult.” – (Parent IDI 18)

Most teachers felt that videos were a good resource for delivering the training. However, they suggested using brochures and pamphlets in those areas that lacked the resources to play videos in schools and larger communities.

“I believe we understand information delivered via video much better than that delivered via pamphlet. Visual learning is much more effective”- (Participant 3, Teachers FGD 1)

“Videos for the schools and brochures/pamphlets for the community.”- (Participant 4, Teachers FGD 3)

### Sub-Theme 3.5: duration, ratio and language of trainer

There was a varied response regarding the duration of the training sessions. However, most participants suggested having small sessions spread over months. They suggested having thirty to sixty minutes long, weekly sessions/classes. The teachers felt shorter classes would keep the students engaged and interested.

“I think it should be given over many weekly sessions for several months. The duration should be short to maintain interest, and make sure that it is not treated as a burden”- (Participant 2, Teacher FGD 1)

“I think 2 classes a week are a must. They should be one hour long”- (Participant 3, Teacher, FGD 2)

“Each class should be thirty to forty minutes long”- (Participant 4, Teacher, FGD 1)

Teachers and students felt there should be frequent refresher training. These could be annual or bi-annual and should have some new and additional knowledge.

“There should be additional knowledge in refresher trainings”- (Participant 3, Students FGD 5)

“There should be annual refreshers”- (Participant 5, Teachers FGD3)

“The refreshers should be conducted every six months”- (Participant 4, Teachers FGD6)

The teachers suggested conducting the training in small group sessions. An ideal situation would be five to ten students per trainer. This would enable a good learning environment, and things would be easier for both the participants and instructors.

“1 instructor for every 5 students would be ideal. A small group would create a better learning environment, especially during the practical component as the instructor would be more comfortable delivering knowledge to a smaller audience and the students will also be more excited to learn/work with more individual attention.” -( Participant 4, Teachers FGD 1).

“I would suggest a maximum of 10 students per instructor as a smaller group would make matters easier for both students and the instructor” (Participant1, Teachers FGD 1)

Most participants suggested using Urdu (National Language) as a mode of instruction. Alternatively, at least it should be bi-lingual in English and Urdu. However, the teachers and students both suggested use of regional languages would be better for rural areas.

“This would depend on the school because English will not be the best decision for many schools. The mother tongue works best, and where English is not working, we can go with Urdu”- (Participant 3, Student FGD 6)

### Theme 4: implementation of national-level lifesaving skills programs

The participants suggested focusing on several action areas if we want to implement an impactful national-level program. All these elements are essential to ensure the successful implementation sustainability of the program. The four sub-themes emerging are: Support from government and senior leadership, integration into the curriculum, legal support and protection, and general public awareness and marketing.

### Sub-Theme 4.1: support from government and senior leadership

Most of the participants felt governmental support was needed to ensure every student gets an opportunity to learn lifesaving skills.

The teachers highlighted a critical issue of socio-economic disparities. They felt learning lifesaving skills was a significant investment, and the government should fund it so that we do not leave any students behind. However, some teachers highlighted the financial constraints for training in all schools.

“The government will have to support the campaign by subsidising these things, else this cannot occur on the large scale”- (Participant 4, Teachers FGD 1)

“We may face potential constraints of financial resources. This would be as only a few schools/students would take interest in a paid course, whereas a free-of-cost course could be difficult to set up.” (Participant 3, Teachers FGD 2)

A parent shared

The provincial health department should be responsible for this. And the financial part should also be the responsibility of the government.” – (Parents IDI 13)

### Sub-Theme 4.2: integration into the curriculum

Most participants felt the lifesaving skills training should be made part of the school and college curriculum. They said it should be made part of the curriculum.

“Schools prepare us for academic life but something like this will prepare us for the life we have ahead of us and god forbid if any tragedy happens, we can prevent it -it should be part of our curriculum”- (Participant 2, Student FGD 3)

Teachers suggested that instead of keeping it as a one-off activity/course, it should be integrated into the curriculum. It should be mandatory training for all the students.

“This will definitely have to be part of the curriculum, as only a stand-alone workshop or a series would not be adequate enough to develop the required level of understanding.”- (Participant 4, Teachers FGD 1)

Further, the teachers suggested a chapter may be added in the science subjects.

“In the book of science, a portion can be added related to the first aid.” (Participant 1, Teachers FGD 06)

When she was a student, one of the teachers recalled that they had basic life support training as part of her school's Physical Education (PE) and recommended a similar course of action.

“I would like to add that when I was in school, basic life support was part of our curriculum. I would suggest similar action, where we introduce this as part of the PE syllabus”. (Participant 5, Teachers FGD 1)

### Sub-Theme 4.3: legal support and protection

Participants talked about changing the legal environment. For example, if the public knew that they would not get legally involved if they helped someone in an emergency, the situation would surely change.

“We assure them if they even mess up, it is in good faith. They are not responsible”- (Participant 3, Students FGD 6)

“A campaign should be done on media so the fear of police from hearts can go. Police will not say anything; we will not get involved in any case” (Participant 4, Teachers FGD 05)

“There's also fear of getting into legal issues and getting involved with police. It won't happen overnight, but as I mentioned earlier awareness campaigns will help. And people would know that they won't get harassed for trying to help out”- (Parent IDI 12)

### Sub-Theme 4.4: general public awareness and marketing

All the participants talked about creating awareness amongst the general public that bystanders can save lives in an emergency. The communities need to be aware that lives can be saved before professional help arrives. Therefore, it is vital to get acceptance by the general public. Different strategies were suggested to get penetration into the communities. This includes; the use of tv shows and advertisements, social media, marketing to create awareness among the masses. Students discussed using the famous morning tv shows and engaging famous personalities such as social media Influencers to reach out to the general public.

“Engage Social Media Influencers”- (Participant 6, Students FGD 07)

“Teach CPR and bleeding control on morning shows”- (Participant 7, Students FGD 05)

Teachers shared their thoughts

“Use sign boards to provide awareness about first aid. You can see social responsibilities on the road. Or upload on social media”- (Participant 5, Teachers FGD 02)

“I think television could be used to provide awareness to the masses first, and then we can have sessions so that people can understand the practical aspect”- (Participant 3, Teachers FGD 1)

## Discussion

This study explored teachers, parents, and students' knowledge, attitudes, and perceptions regarding implementing a school-based lifesaving skills program and identified potential barriers and facilitators in a low-resource country. The most commonly cited barriers was bystanders’ lack of knowledge about lifesaving skills hampering their appropriate engagement and response during an emergency. This barrier is similar to other studies where the bystanders did not have good knowledge of CPR and other lifesaving skills [22]. This coupled with the fear of hurting a victim of cardiac arrest due to incorrect technique can be addressed through existing training courses. Our findings are consistent with studies from other countries reporting a lack of awareness and knowledge regarding bystander response [23–25]; the fear of causing more harm has been reported from multiple settings around the world [15, 18, 26, 27].

Most participants identified the fear of harassment by police and the medico-legal system.as a barrier and cited lack of laws safeguarding lay rescuers in Pakistan. This finding is similar to studies from China and Scotland, where bystanders feared being caught up in a legal dispute as a barrier [17, 28]. Public perception that CPR and other life saving skills should only be performed by medical professionals are further reinforced by policies discouraging bystander engagement. Many high-income countries have legislation and policies protecting bystanders who attempt to give lifesaving assistance to those in need. Countries such as Australia, Belgium, Canada, Finland, US and India provide variation of language and expectation and could be a good starting point for country like Pakistan [29].

In conservative societies such as Pakistan, hesitation to physically touch victims of opposite sex is not surprising. As a result, unfortunately, women victims of cardiac arrest or injuries are less likely to receive lifesaving care. Normative changes, perhaps using political and religious leadership is a long-term solution. In addition, it is critical that women in society are trained in the lifesaving skills leading to improved access of female victims to CPR and other similar interventions. This finding is similar to the Chinese study, where the college students expressed embarrassment in helping opposite gender victims [17].

Coinciding with other studies, almost all the participants showed a positive attitude toward learning lifesaving skills in schools [19]. They felt the students were physically strong enough to learn and implement these skills. However, they may lack confidence, which can be overcome by providing adequate training [19]. Few participants shared concerns that this training may affect their studies and overburden them. However, most suggested that conducting these training in free periods would be acceptable [30].

Our findings show great support from all participants for implementing lifesaving skills training programs in schools. They suggested starting training early in life, commencing in the grade 9th and secondary school. This is in line with WHO's recommendation of teaching CPR to school children aged 12 years and above. In addition, most participants felt more comfortable with medical professionals as the instructors for life-skills training in school [31]. This suggestion is similar to other studies where the health professionals were preferred instructors [31, 32].

Furthermore, the teachers felt that they will not be able to deliver training given the existing work load. Potential trainers identified by participants included physical education teachers, science teachers, or new teachers. The solution would require school leadership to assess individual teacher’s workload. Nonetheless, this finding aligns with previous studies where most teachers were unwilling and opposed to teaching themselves [27, 31, 32].

Most participants suggested face-to-face sessions, lectures for teaching the theory, and practical/hands-on drills for the skills session. However, the participants suggested using radio, pamphlets, or brochures for the rural/underprivileged schools lacking necessities such as electricity and internet. There are varied instruction approaches in the literature, ranging from conventional to innovations [30]. Most participants suggested having small sessions spread over months, with annual/biannual refreshers [32]. It was suggested to keep the training in the National language Urdu or English/Urdu. It was suggested to conduct the training in regional languages for the rural areas. This is an essential consideration while developing a nationwide curriculum.

Most participants felt governmental support was needed to train students across Pakistan. This is in line with other studies where government, school, and other professional bodies' support was felt essential for the successful implementation of the program [30]. In addition, almost all the participants emphasized the importance of lifesaving skills and suggested integrating it into the curriculum [30]. This will be essential in systematically teaching all schoolchildren and a step towards achieving the goal of building a nation of lifesavers.

## Limitations

Our study has some limitations. We included schools from Karachi, which is a metropolitan city. Schools in smaller cities and rural areas may have different perspectives and barriers due to cultural and socio-economic differences. Also, our sampling represented both the government and private schools. We decided to recruit more participants from private schools, given the larger number of these schools in Karachi and the greater willingness of private school leadership and teachers to participate. Even with the uneven distribution of school types, we do not anticipate significant differences in the perceptions regarding lifesaving skills training between government and private schools.

## Conclusion

This study found that bystanders' main barriers to performing lifesaving skills are lack of knowledge, fear of legal repercussions, fear of hurting the patient, and gender bias. This study indicates that the participants had a positive and supportive attitude toward implementing lifesaving skills training in schools. It was suggested to start student training in the early teenage years, preferred medical staff as trainers, and suggested frequent small sessions in English/Urdu both or Urdu language, and training via theory and practical hands-on drills. Furthermore, it was proposed that the trainings should be integrated into the school curriculum to make it sustainable. The government needs to support the program and make the legal environment more conducive for the bystanders to systematically train all the students in schools and colleges.

## Data Availability

The data that support the findings of this study are available from the corresponding author upon reasonable request.

## References

[1] Porzer M, Mrazkova E, Homza M, Janout V (2017). Out-of-hospital cardiac arrest. Biomedical Papers.

[2] Böttiger BW, van Aken H (2015). Training children in cardiopulmonary resuscitation worldwide. The Lancet.

[3] Nichol G (2008). Regional variation in out-of-hospital cardiac arrest incidence and outcome. JAMA.

[4] Pan J, Zhu JY, Kee HS, Zhang Q, Lu YQ (2015). A review of compression, ventilation, defibrillation, drug treatment, and targeted temperature management in cardiopulmonary resuscitation. Chin Med J.

[5] Tanigawa K, Iwami T, Nishiyama C, Nonogi H, Kawamura T (2011). Are trained individuals more likely to perform bystander CPR? An observational study. Resuscitation.

[6] Swor R (2006). CPR Training and CPR Performance: Do CPR-trained Bystanders Perform CPR?. Acad Emerg Med.

[7] Böttiger BW, van Aken H (2015). Kids save lives–Training school children in cardiopulmonary resuscitation worldwide is now endorsed by the World Health Organization (WHO). Resuscitation.

[8] Chamberlain DA, Hazinski MF (2003). Education in resuscitation: an ILCOR symposium. Circulation.

[9] Lockey AS, Georgiou M (2013). Children can save lives. Resuscitation.

[10] Neumar RW (2015). American Heart Association Response to the 2015 Institute of Medicine Report on Strategies to Improve Cardiac Arrest Survival. Circulation.

[11] Abelairas-Gómez C, Rodríguez-Núñez A, Casillas-Cabana M, Romo-Pérez V, Barcala-Furelos R (2014). Schoolchildren as life savers: at what age do they become strong enough?. Resuscitation.

[12] Hill K, Mohan C, Stevenson M, McCluskey D (2009). Objective assessment of cardiopulmonary resuscitation skills of 10–11-year-old schoolchildren using two different external chest compression to ventilation ratios. Resuscitation.

[13] Shi HT, Ge JB (2016). Improving public defibrillator use in China. The Lancet.

[14] Chen M (2017). Public knowledge and attitudes towards bystander cardiopulmonary resuscitation in China. BioMed Res Int.

[15] Andrews, Tiana & Price, Luke & Mills, Brennen & Holmes, Lisa. (2018). Young adults’ perception of mandatory CPR training in Australian high schools: A qualitative investigation. Australasian Journal of Paramedicine. 2018;15. 10.33151/ajp.15.2.577.

[16] Zinckernagel L, Malta Hansen C, Rod MH (2016). What are the barriers to implementation of cardiopulmonary resuscitation training in secondary schools? A qualitative study. BMJ Open.

[17] Lu C, Jin Y, Meng F, Wang Y, Shi X, Ma W, Chen J, Zhang Y, Wang W, Xing Q (2016). An exploration of attitudes toward bystander cardiopulmonary resuscitation in university students in Tianjin, China: a survey. Int Emerg Nurs.

[18] Anto-Ocrah M, Maxwell N, Cushman J (2020). Public knowledge and attitudes towards bystander cardiopulmonary resuscitation (CPR) in Ghana, West Africa. Int J Emerg Med.

[19] Shams A, Raad M, Chams N, Chams S, Bachir R, El Sayed MJ (2016). Community involvement in out of hospital cardiac arrest: a cross-sectional study assessing cardiopulmonary resuscitation awareness and barriers among the Lebanese youth. Medicine (Baltimore).

[20] IPRI - Islamabad Policy Research Institute. 2022. Education System of Pakistan: Issues, Problems and Solutions - IPRI - Islamabad Policy Research Institute. [online] Available at: <https://ipripak.org/education-system-of-pakistan-issues-problems-and-solutions/> [Accessed 8 May 2022].

[21] Virginia Braun & Victoria Clarke. (2006). Using thematic analysis in psychology. Qual Res Psychol. 3:2, 77 101. 10.1191/1478088706qp063oa

[22] Al Harbi N, Afifi A, Alateeq M, Tourkmani A, Alharbi T, Albattal S. Awareness of basic life support and cardiopulmonary resuscitation among female secondary school students in government schools in Riyadh city, KSA. J Family Med Prim Care. 2018;7(6):1493–1500. 10.4103/jfmpc.jfmpc_21_18. PMID: 30613548. 10.4103/jfmpc.jfmpc_21_18 PMC6293950 30613548

[23] Adewale BA, Aigbonoga DE, Akintayo AD, Aremu PS, Azeez OA, Olawuwo SD, Adeleke JD, Kazeem OS, Okojie E, Oguntoye RA. Awareness and attitude of final year students towards the learning and practice of cardiopulmonary resuscitation at the University of Ibadan in Nigeria. Afr J Emerg Med. 2021;11(1):182–187. 10.1016/j.afjem.2020.09.019. Epub 2020 Oct 19. PMID: 33101886; PMCID: PMC7571441. 10.1016/j.afjem.2020.09.019 PMC7571441 33101886

[24] Chilappa R, Waxman MJ (2021). Basic life support awareness and knowledge in high school students. Kans J Med.

[25] Al Harbi N, Afifi A, Alateeq M, Tourkmani A, Alharbi T, Albattal S. Awareness of basic life support and cardiopulmonary resuscitation among female secondary school students in government schools in Riyadh city, KSA. J Family Med Prim Care. 2018;7(6):1493–1500. 10.4103/jfmpc.jfmpc_21_18. PMID: 30613548; PMCID: PMC6293950. 10.4103/jfmpc.jfmpc_21_18 PMC6293950 30613548

[26] Petrić J, Malički M, Marković D, Meštrović J. Students’ and parents’ attitudes toward basic life support training in primary schools. Croat Med J. 2013;54(4):376–80. 10.3325/cmj.2013.54.376. 10.3325/cmj.2013.54.376 PMC3760662 23986279

[27] Lieven De Smedt, Catheline Depuydt, Eva Vekeman, Peter De Paepe, Koenraad G. Monsieurs, Martin Valcke & Nicolas Mpotos. Awareness and willingness to perform CPR: a survey amongst Flemish schoolchildren, teachers and principals. Acta Clinica Belgica. 2018. 10.1080/17843286.2018.1482087. 10.1080/17843286.2018.1482087 29874976

[28] Dobbie F, Uny I, Eadie D, Duncan E, Stead M, Bauld L (2020). Barriers to bystander CPR in deprived communities: findings from a qualitative study. PLoS ONE.

[29] Good samaritan rights and duties of the hospitals/police https://morth-roadsafety.nic.in/. Available at: http://www.morth-roadsafety.nic.in/pdf/Good-Samaritan.pdf (Accessed: 2 Feb 2023).

[30] Reveruzzi B, Buckley L, Sheehan M (2016). School-based first aid training programs: a systematic review. J Sch Health.

[31] Mpotos, Nicolas & Vekeman, Eva & Monsieurs, Koenraad & Derese, Anselme & Valcke, Martin. Knowledge and willingness to teach cardiopulmonary resuscitation: A survey amongst 4273 teachers. Resuscitation. 2013;84. 10.1016/j.resuscitation.2013.01.023. 10.1016/j.resuscitation.2013.01.023 23376584

[32] Miró O, Jiménez-Fábrega X, Espigol G, Culla A, Escalada-Roig X, Díaz N, Salvador J, Abad J, Sánchez M (2006). Teaching basic life support to 12–16 year olds in Barcelona schools: views of head teachers. Resuscitation.

